# Hypoxia-Responsive Polymeric Micelles for Enhancing Cancer Treatment

**DOI:** 10.3389/fchem.2020.00742

**Published:** 2020-09-04

**Authors:** Huayang Feng, Dandan Chu, Fan Yang, Zhanrong Li, Bingbing Fan, Lin Jin, Jingguo Li

**Affiliations:** ^1^Henan Provincial People's Hospital, Zhengzhou University People's Hospital, Zhengzhou, China; ^2^School of Materials Science and Engineering, Zhengzhou University, Zhengzhou, China

**Keywords:** hypoxia-responsive, polymeric micelles, cancer treatment, doxorubicin, drug delivery

## Abstract

Polymeric drug vectors have shown great potentials in cancer therapy. However, intelligent controlled release of drugs has become a major challenge in nanomedicine research. Hypoxia-responsive polymeric micelles have received widespread attention in recent years due to the inherent hypoxic state of tumor tissue. In this study, a novel diblock polymer consisting of polyethylene glycol and poly[glutamic acid (3-(2-nitro-imidazolyl)-propyl)] was synthesized and self-assembled into hypoxia-responsive polymeric micelles for the controlled release of doxorubicin (DOX). The cell experiments demonstrated that DOX-loaded micelles had a stronger killing capacity on tumor cells under hypoxic conditions, while the blank micelles had good biocompatibility. All the experiments indicate that our hypoxia-responsive polymeric micelles have a great potential for enhanced cancer treatment.

**Graphical Abstract:**
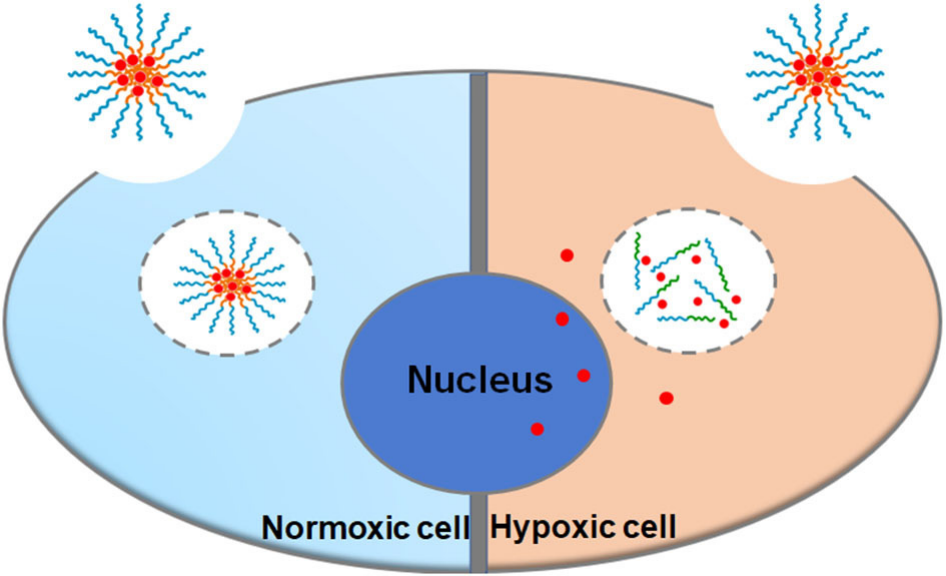
A hypoxia-responsive polymer micelle encapsulated with DOX was developed for therapy of cancer. *in vitro* studies demonstrated the enhanced anticancer effect of the nanomicelles.

## Introduction

Cancer is a disease that endangers human life. About 9.6 million people died because of cancer in 2018 according to American Cancer Society (Bray et al., 2018). Therefore, the development of more effective methods for the treatment of cancer is of great significance for promoting human health. At present, the main treatments for cancer include chemotherapy, radiotherapy, and surgery. Among them, surgery and radiation therapy are local treatments, which can only kill local tumor cells. Chemotherapy is a whole-body treatment method, which can kill not only native tumor cells but also metastatic cancer cells, and has a systemic therapeutic effect (Krishnan and Rajasekaran, 2014). However, most chemotherapeutic drugs such as DOX generally have shortcomings like large toxic side effects and short drug effects, which seriously affect the clinical tumor treatment effect (Feng et al., 2018; Pugazhendhi et al., 2018).

To solve these problems, researchers have developed many nanoscale drug delivery systems to improve the anticancer efficacy of drugs (Li et al., 2014a,b; Li et al., 2016b; Chen et al., 2018; Gao et al., 2019; Guo et al., 2020; Ma et al., 2020). Among many drug delivery vehicles, polymeric micelles have attracted more and more researchers' attention because of their low biological toxicity, high stability, and high drug loading capacity (Li et al., 2016a; Huang et al., 2018; Li et al., 2020a,b). Polymer micelles are usually composed of a hydrophilic shell and a hydrophobic core, which are usually self-assembled from amphiphilic block polymers in water with a size varying from 10 to 200 nm. The above structure of the polymeric micelles gives them the following advantages: (1) it can improve the water solubility of many hydrophobic drugs; (2) it can extend the circulation time of the drugs in the body through reducing the clearance of the drug by the kidney; (3) it can increase the concentration of drugs in tumor tissues through enhanced penetration and retention effects (EPR effect), also known as passive targeting (Matsumura and Maeda, 1986; Danhier et al., 2010).

More and more researchers have concentrated on stimuli-responsive polymeric nanocarriers because of their good application prospects in drug delivery and intelligent controlled release of drugs (Dong et al., 2016; Kamaly et al., 2016; Gao and Dong, 2018; Hu et al., 2019; Song et al., 2019). Among them, hypoxia-responsive polymeric nanocarriers are a new class of stimulus-responsive carriers that have been studied recently (Liu et al., 2015; Dong et al., 2020). Due to the strong growth of the tumor tissue and the relatively insufficient blood supply, the tumor tissues are usually in a hypoxic state compared with the normal tissues. Researchers have developed a variety of hypoxia-responsive carriers based on the characteristics of tumor tissue hypoxia. The carriers can disintegrate or deform to release drugs in hypoxic tumor tissues, but will not disintegrate or deform in normal tissues, thereby improving drug efficacy and reducing toxic side effects. Among many compounds with hypoxia responsiveness, 2-nitroimidazole has been mostly studied (Thambi et al., 2014). 2-Nitroimidazole (NI) is hydrophobic, and it could be reduced to hydrophilic aminoimidazole easily by enzymes in the body under hypoxic conditions, which can be used as an important component of hypoxia-responsive carriers.

**Scheme 1:**
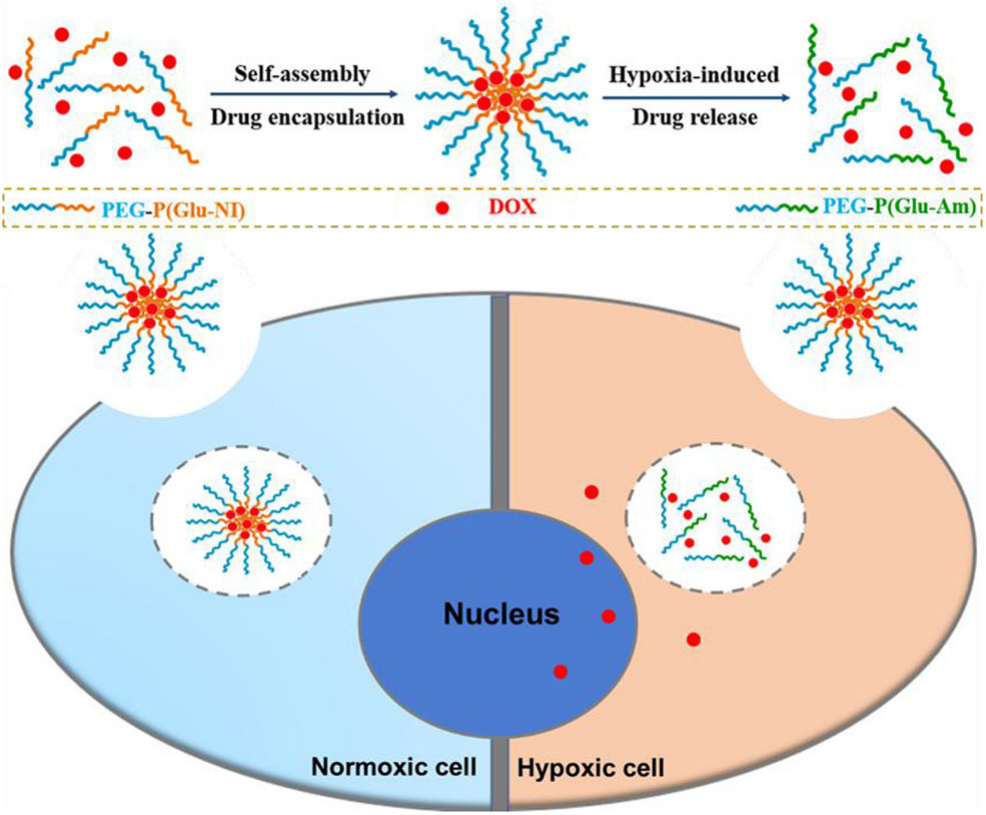
Schematic of fabrication and hypoxia-sensitive drug release process.

Herein, a novel diblock hypoxia-responsive polymer consisting of polyethylene glycol(PEG) and poly(glutamic acid (3-(2-nitro-imidazolyl)-propyl))(P(LGlu-NI)), abbreviated as PEGN, is synthesized and self-assembled to hypoxia-responsive micelles for the controlled release of DOX (Scheme 1). The PEG block served as a hydrophilic shell, and the P(LGlu-NI) block is used as a hypoxia-responsive hydrophobic core to support DOX. Under hypoxic conditions, the hydrophobic nitroimidazole could turn to the hydrophilic aminoimidazole, which could make the micelle disintegrated or deformed, thereby releasing DOX quickly. The anticancer activity of DOX-loaded micelles was examined in human breast adenocarcinoma MCF-7 cells.

## Experimental

### Materials

a-Methoxy-3-amino poly (ethylene glycol) (mPEG-NH_2_, Mn = 2 kDa) was purchased from Ponsure Biotech (Shanghai, China). 1-Chloro-3-hydroxypropane (Macklin, China), DL-glutamic acid (Macklin, China), 2-nitroimidazole (Accela, China), and doxorubicin hydrochloride (Melone Pharma, China) were used as received. A dialysis bag (Mw cutoff: 3.5 kDa) was purchased from Shanghai Green Bird Technology Development Co., Ltd. (Shanghai, China). Anhydrous *N, N*-dimethylformamide (DMF) was purchased from JK chemical (Beijing, China). Menthol, diethyl ether, petroleum ether, and ethyl acetate were purchased from Zhengzhou Yinfeng Reagent Co., Ltd. (Zhengzhou, China). Petroleum ether and ethyl acetate were dried over CaH_2_ and then distilled under ambient pressure.

MCF-7 cells were obtained from the Kunming cell library of the Chinese Academy of Sciences. MEM medium was purchased from HyClone (America). Phosphate-buffered saline (PBS) and 1% penicillin–streptomycin double antibody were purchased from Solarbio (Shanghai, China). Fetal bovine serum (FBS) was purchased from SeraPro (Germany). Trypsin–EDTA (0.25%) solution was purchased from Gibco (America). Cell Counting Kit-8 (CCK-8) was purchased from Dojindo.

### Synthesis of Diblock Copolymers (PEGN)

#### Synthesis of Glutamic Acid (3-Chloropropyl) (LGlu-Cl)

11 g glutamic acid was dissolved in 100 mL of 3-chloro-1-propanol, and then 10 mL trimethylchlorosilane was added into the above system. The reaction was allowed to proceed at room temperature for 5 days. After being transparent, the reaction solution was precipitated into a large amount of diethyl ether, filtered, washed three times with diethyl ether, and finally dried for 24 h under vacuum.

#### Synthesis of N-Carboxyanhydride of Glutamic Acid (3-Chloropropyl) (LGlu-Cl-NCA)

5.8 g LGlu-Cl was dissolved in 200 mL of ethyl acetate, and then the mixture was stirred at 70°C. After the mixture started to reflux, 3.2 g of triphosgene dissolved in 20 mL of ethyl acetate was added into the above mixture slowly. The reaction was allowed to proceed at 70°C for 4 h. After being transparent, the reaction solution was cooled in a refrigerator at −20°C. The reaction solution was washed with cold saturated Na_2_CO_3_ and NaCl solution and then dried with anhydrous MgSO_4_. After being concentrated using a rotary evaporator, the solution was precipitated into a large amount of petroleum ether, filtered, and recrystallized with petroleum ether and ethyl acetate for the subsequent ring-opening polymerization reaction.

#### Synthesis of PEG-b-P(LGlu-Cl)

0.7 g of PEG-NH_2_ was dissolved into 20 mL of anhydrous DMF. Then, 2.6 g (LGlu-Cl)-NCA was added into the above solution under the protection of nitrogen, and the reaction was allowed to proceed at 40°C for 3 days. The above system was transferred to a dialysis bag (Mw cutoff: 3.5 kDa) and dialyzed against pure water for 3 d and lyophilized to obtain a white powder, which was the target product (Mn = 4.9 kDa, calculated from the 1H NMR spectrum, mPEG_45_-b-P (LGlu-Cl)_14_).

#### Synthesis of PEG-b-P(LGlu-NI) (PEGN)

294.9 mg NI and 352.7 mg K_2_CO_3_ were dissolved in 10 mL of DMF and stirred for 10 min at room temperature. 711 mg PEG-b-P(LGlu-Cl) and 60 mg NaI were dissolved in 10 mL of DMF and then slowly added into the above solution. The reaction was allowed to proceed for 72 h at 80°C. The above system was dialyzed against deionized water for 3 days and lyophilized to obtain PEGN.

### Preparation of the Micelles

30.0 mg of PEGN and 3.0 mg DOX·HCl were dissolved in 3 mL DMSO, then 5 μL of triethylamine was added to the above mixture to turn hydrophilic DOX·HCl into hydrophobic DOX. The above solution was added dropwise to 30 mL of deionized water under ultrasonic conditions. Then, the above solution was transferred into a dialysis bag and dialyzed for 1 days in deionized water to remove DMSO and unencapsulated DOX. After the dialysis, the solution was filtered with a 450-nm filter to remove large particles, and the solution was concentrated and washed three times using an ultrafiltration tube (MWCO = 100,000 Da) with physiological saline to obtain DOX-loaded micelles (PEGN/DOX). The preparation of blank micelles (PEGN bm) is the same as that of DOX-loaded micelles, except that DOX is not added during the preparation process.

### Characterization of the Polymers and Nanoparticles

^1^H NMR spectra of the monomer and polymers were obtained on a Bruker AVANCE II 400-MHz spectrometer using DMSO-d_6_ as the solvent. Fourier transform infrared (FTIR) spectral studies were carried out using an IS10 670 FTIR spectrometer, and all the samples were compressed into pellets with KBr before being tested. The hydrodynamic sizes of the micelles were determined using a Nano ZS90 dynamic light scattering (DLS) equipment. The data was collected on an auto-correlator with a detection angle of scattered light at 90°. Each sample was measured three times, and the results were obtained by the average of the collected data. UV absorption of blank micelles under normal and hypoxic conditions was detected by an ultraviolet-visible spectrophotometer.

### Loading Content of DOX

A UV-Vis spectrophotometer was used to determine the drug loading content of DOX. Firstly, the DOX-loaded PEGN micelle solution was lyophilized, weighed, and dissolved in DMSO. Then, the DOX concentration was quantified by measuring the absorbance of DOX at 480 nm according to a standard curve we established in advance.

### Confocal Laser Scanning Microscopy (CLSM)

The cellular uptake of micelles in MCF-7 cells for different groups was analyzed on a confocal laser scanning microscope (Nikon C1si, Japanese). For the normoxic group, MCF-7 cells were incubated overnight in 6-well plates at 37°C and then incubated with PEGN/DOX micelles for 2 h. The cells were washed with PBS twice, and the nuclei were stained with Hoechst 33342 solution for 10 min. Finally, MCF-7 cells were analyzed and observed under CLSM. The excitation and emission wavelengths of DOX and Hoechst are 488 nm and 590 nm, 350 nm, and 460 nm, respectively. Except that the MEM medium containing 100 μM CoCl_2_ was used to simulate the *in vivo* hypoxic environment, the experimental steps for the hypoxic group were the same as the above steps.

### Cell Viability Assay

MCF-7 cells were seeded in 96-well plates with MEM medium containing 100 μM CoCl_2_ and incubated at 37°C under 5% CO_2_ atmosphere for 24 h. Then, the cells were separately incubated with blank and DOX-loaded micelles at various concentrations for 48 h. After the medium in each well was replaced with 100 μL fresh medium containing 10 μL CCK-8 solutions, the cells were incubated for another 4 h. After vibration for 10 min, the absorbance at 450 nm was analyzed with an Enzyme Labeler (PerkinElmer EnVision). Cell viability was calculated by comparing absorbance with the negative control. All experiments were conducted in triplicate.

### Cell Apoptosis by TUNEL

The cell apoptosis rates with PEGN/DOX in normoxic and hypoxic groups were detected by TUNEL assay. For the normoxic group, MCF-7 cells were seeded into 6-well plates and cultured overnight. Then, the cells were incubated with PEGN/DOX for 24 h. Then, the cells were fixed with 4% paraformaldehyde (200 μL) for 20 min and then washed three times with PBS. Furthermore, the cells were treated with 100 μL of 1% Triton X-100 for 5 min and then washed three times with PBS. After adding 50 μL of TdT enzyme solution to each sample, the samples were washed three times with PBS. Then, the samples were treated with 50 μL of streptavidin–fluorescein solution for 30 min and washed three times with PBS. Then, the nuclei were stained with Hoechst 33342 solution for 10 min and washed with PBS for three times. At last, the samples were observed with a fluorescence microscope (Nikon ECLIPSE 80i, Japan). Except that the MEM medium containing 100 μM CoCl_2_ was used to simulate the hypoxic environment *in vivo*, the experimental steps for the hypoxic group were the same as the above steps.

## Results and Discussion

### Polymer Synthesis and Characterization

**Scheme 2:**
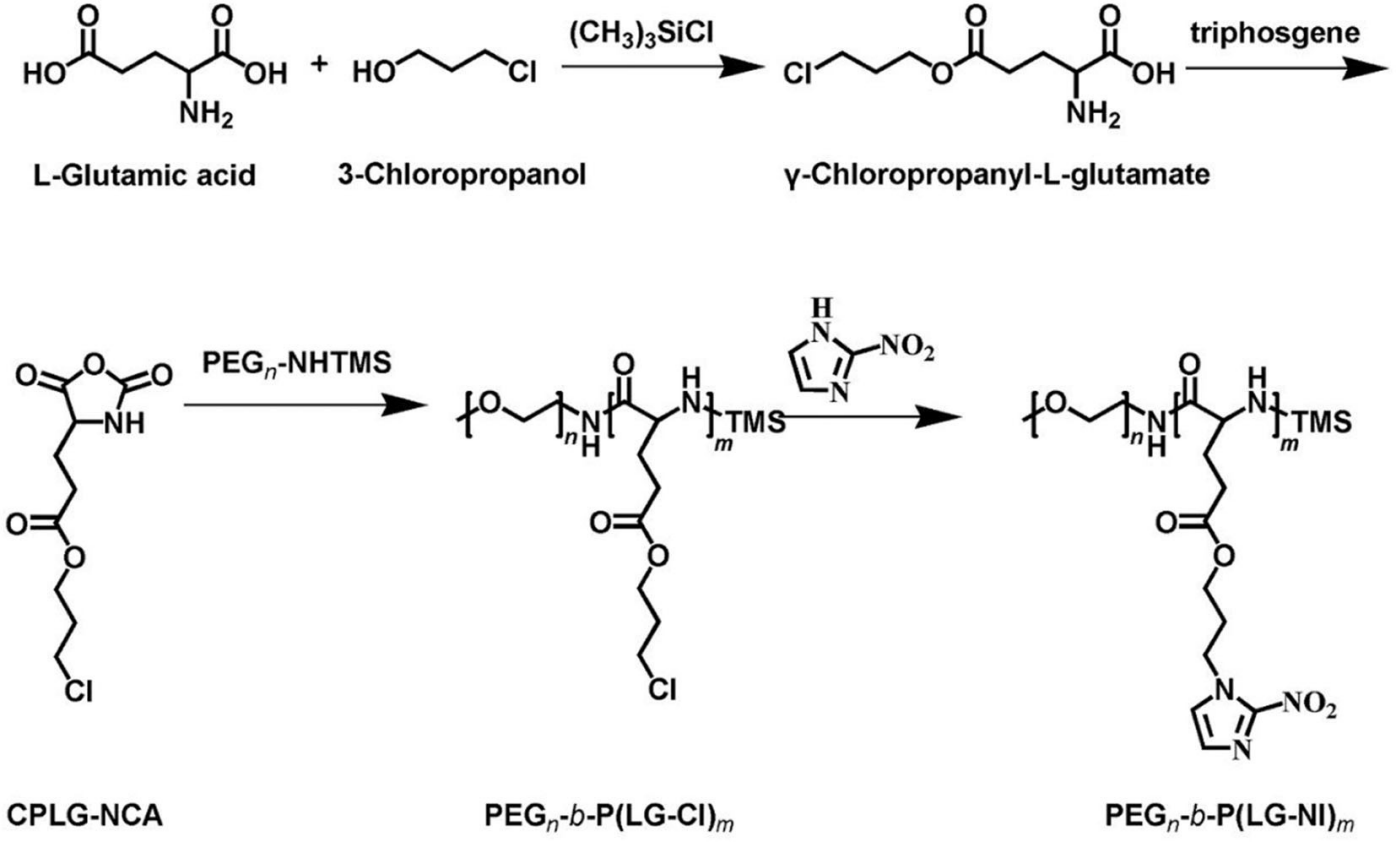
The synthesis approach of copolymer PEGN.

**Figure 1:**
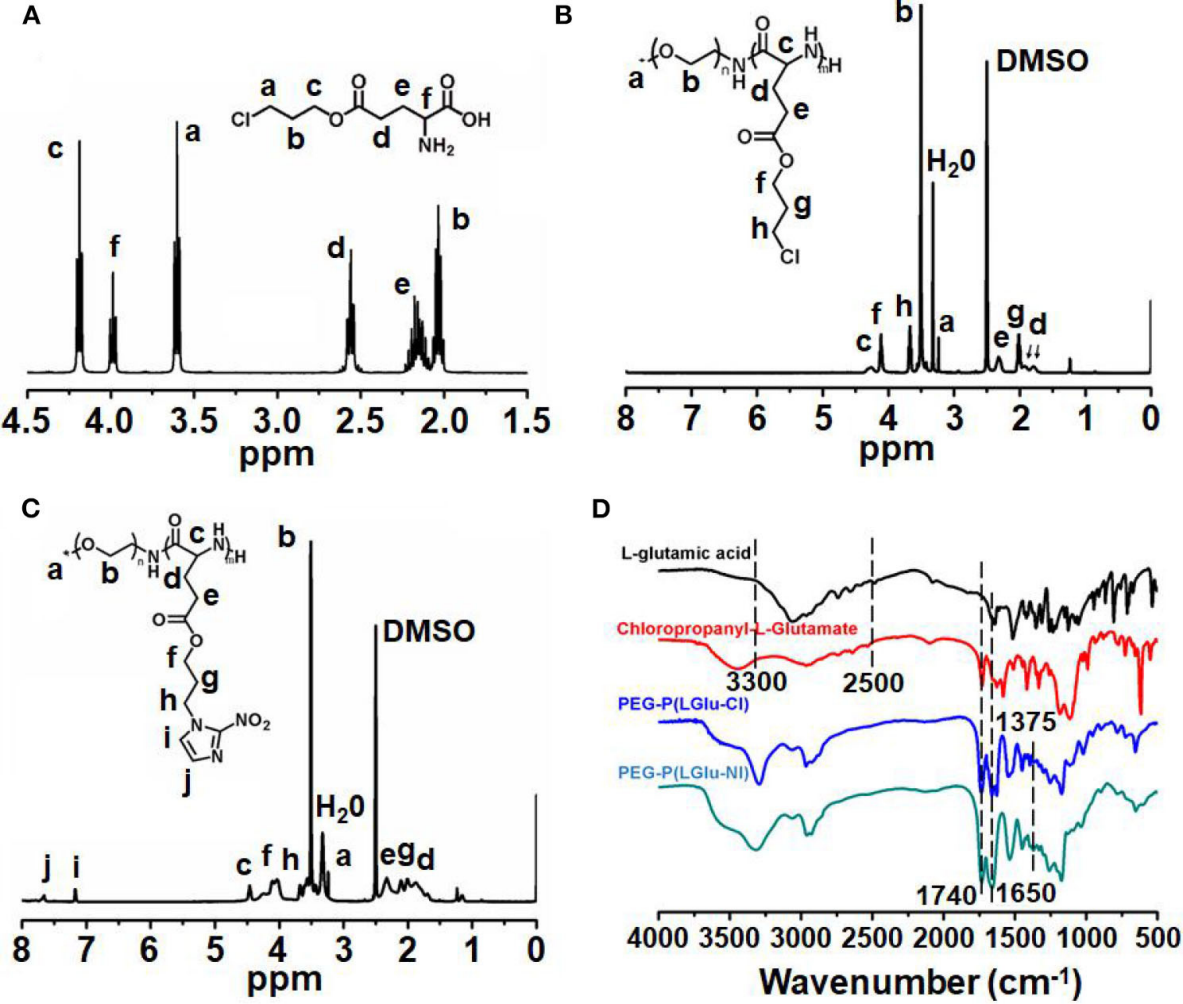
^1^H-NMR spectra of **(A)** LGlu-Cl and **(B)** PEG-b-P(LGlu-Cl) and **(C)** PEGN; **(D)** FTIR spectra of PEGN and its precursors.

The synthetic approach of PEG-b-P(LGlu-NI) is illustrated in Scheme 2. Moreover, the chemical structure of monomer and polymer was verified by ^1^H NMR and FTIR analyses (Figure 1). Figure 1A shows the ^1^H NMR spectra of LGlu-Cl. The peaks are assigned as follows: 4.19 ppm (ClCH_2_CH_2_C***H***_2_O–), 4.00 ppm (NH_2_ (–CH_2_-)C***H***–CO–), 3.60 ppm (ClC***H***_2_CH_2_CH_2_-), 2.56 ppm (–COC***H***_2_CH_2_-), 2.09–2.21 ppm (–COCH_2_C***H***_2_-), and 2.02 ppm (ClCH_2_C***H***_2_CH_2_-), indicating the successful synthesis of the target product. The ^1^H NMR spectra of PEG-b-P(LGlu-Cl) and PEG-b-P(LGlu-NI) are shown in Figures 1B,C. The proton chemical shifts in the ^1^H NMR spectrum of PEG-b-P(LGlu-Cl) are 3.50 ppm (–OC***H***_**2**_C***H***_**2**_–), 3.28 ppm (C***H***_3_-OCH_2_CH_2_-), 4.28 ppm (–NH(–CH_2_-)C***H***–CO–), 4.10 ppm (ClCH_2_CH_2_C***H***_2_O–), 3.66 ppm (ClC***H***_2_CH_2_CH_2_-), 2.34 ppm (–COC***H***_2_CH_2_-), 2.02 ppm (ClCH_2_C***H***_2_CH_2_-), and 1.75–1.91 ppm (–NH(–C***H***_2_-)CH–CO–). According to the ratio of integral values of glutamic acid block signals to the PEG block signals, the polymerization degree of LGlu-Cl moiety is 14. After the ammonolysis reaction, the appearance of the two characteristic signals of nitroimidazole at 7.65 ppm (–NC***H***CHN–) and 7.20 ppm (–NCHC***H***N–) indicate the successful synthesis of the final product PEGN (Figure 1C). Figure 1D shows the FTIR spectrum of products; the appearance of a characteristic peak of nitroimidazole around 1,375 cm^−1^ confirms the formation of PEGN. The presence of characteristic peaks of ester and amide bonds at 1,740 and 1,650 cm^−1^ indicates that the polymer structure is complete before and after the reaction, and there was no degradation.

### Preparation and Characterization of Micelles

**Figure 2:**
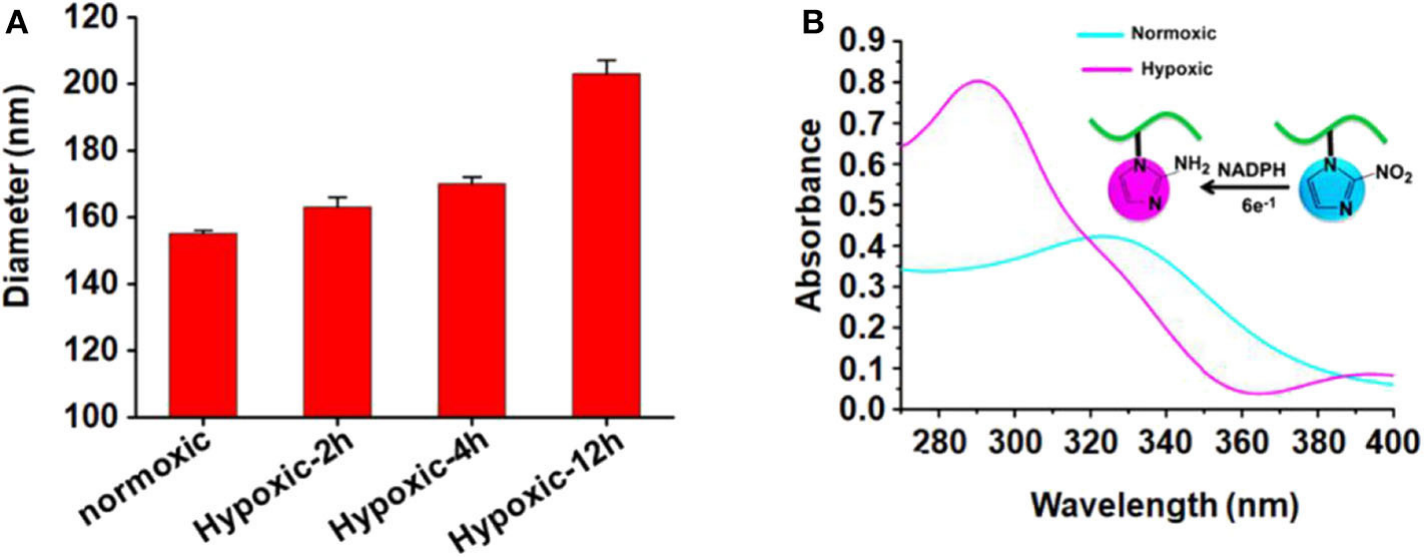
**(A)** Mean particle size of blank micelles with prolonged hypoxic treatment time; **(B)** UV absorption of PEGN bm at hypoxic and normoxic conditions.

The size of micelles was evaluated using DLS. The mean diameter of the blank micelle and DOX-loaded micelle is 155 and 168 nm, respectively. The reason for the change of particle size may be that the loading of DOX increases the volume of the hydrophobic core, which increases the overall particle size of the micelles. Figure 2A shows the change of the average particle size of blank micelles with prolonged hypoxic treatment time. Under hypoxic conditions, the mean diameter of blank micelles changed from 155 to 203 nm, probably because nitroimidazole was converted to aminoimidazole under hypoxic conditions, which reduced the hydrophobicity of the hydrophobic core and led to internal aggregation force weakening, thereby increasing the volume of the micelles. By calculation with previously established calibration curves, the drug loading content of the micelles was 3.99% (encapsulation efficiency of 41.57%). Figure 2B is the UV absorption curve of a blank micelle under hypoxic and normal conditions. Under normal conditions, there is only very weak UV absorption at 327 nm, but a strong UV absorption peak appears at 290 nm under hypoxic conditions, indicating that the nitroimidazole group was converted into aminoimidazole under hypoxic conditions, which is consistent with the reported literature (Thambi et al., 2014).

### Cell Uptake and Intracellular Distribution

**Figure 3:**
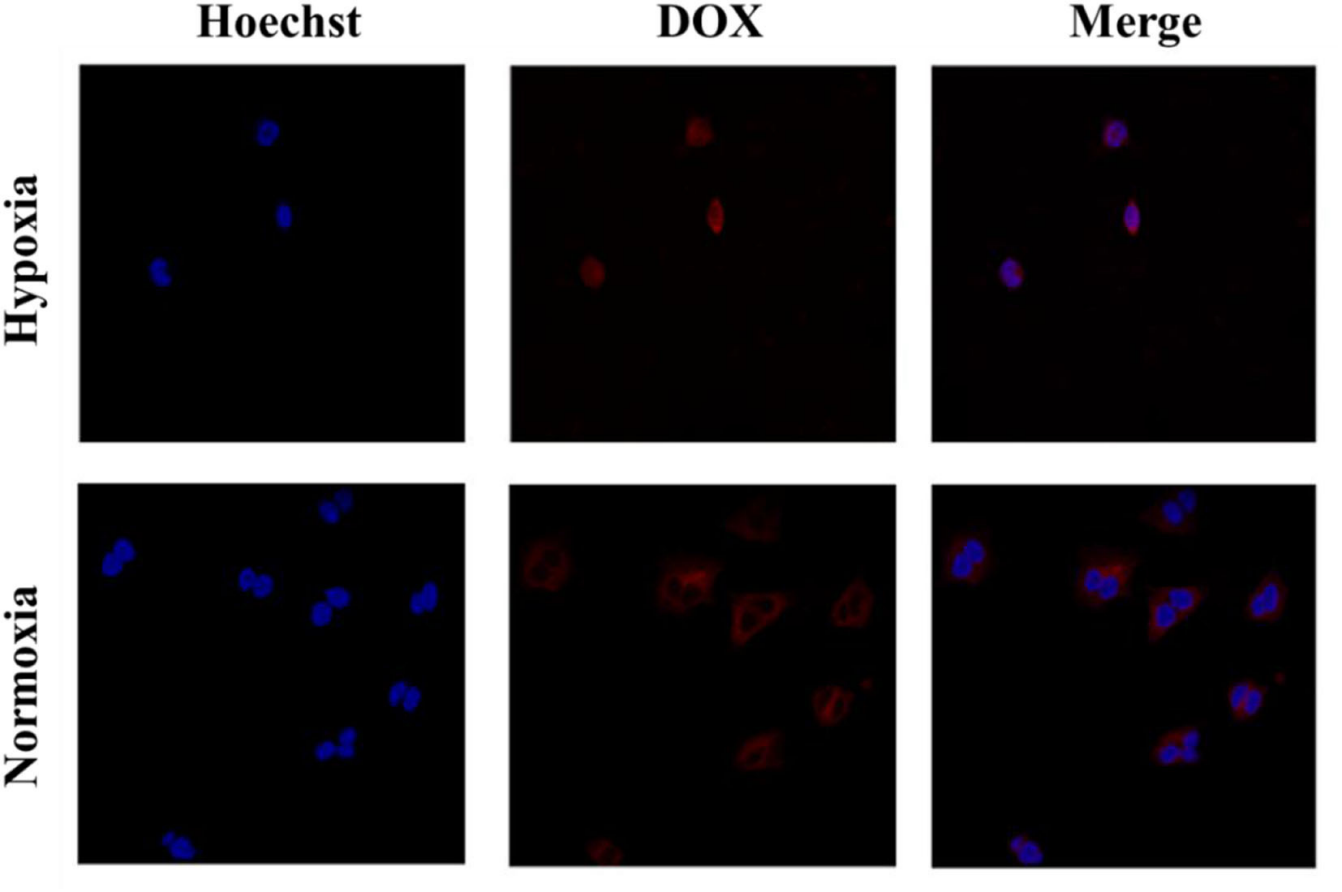
Cellular uptake of PEGN/DOX after being cultured with MCF-7 cells for 2 h under hypoxic conditions and normoxic conditions.

Cell uptake and intracellular distribution of the DOX-loaded micelles were evaluated with CLSM. The results were obtained in MCF-7 cells. The red fluorescence indicates the position of DOX, and the blue fluorescence indicates the position of the nucleus stained with Hoechst, and the purple fluorescence is obtained by the merge of red and blue fluorescence. Figure 3 shows the situation of DOX delivered into cancer cells under hypoxic conditions and normoxic conditions. After incubation with the micelles for 2 h under hypoxic conditions, intense purple fluorescence was observed in the nucleus, indicating that most of the DOX were delivered into the nucleus. However, after incubation with the micelles for 2 h under normoxic conditions, DOX was still mainly distributed in the cytoplasm. These results show that micelles can respond to hypoxia conditions and deliver drugs into the nucleus of tumor cells more quickly under hypoxic conditions.

### Cytotoxicity

**Figure 4:**
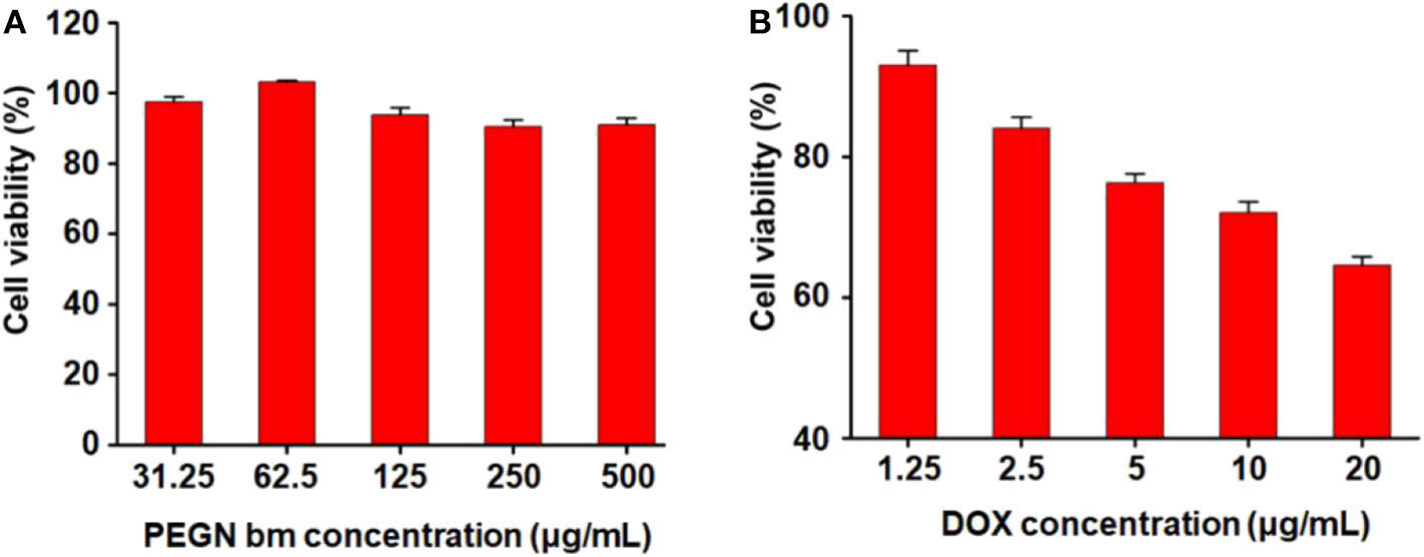
Cytotoxicity of **(A)** PEGN bm and **(B)** PEGN/DOX.

The cytotoxicity of micelles was determined in MCF-7 cells by CCK-8 assay. Figure 4A shows the cytotoxicity of blank micelles under hypoxic conditions. With the increase in the blank micelle concentration, the cell survival rate did not change much (all remained above 90%), indicating that the carrier has very good biological safety. Figure 4B shows the cytotoxicity of DOX-loaded micelles under hypoxic conditions. As the concentration of drug-loaded micelles increased, the survival rate of tumor cells decreased rapidly. When the concentration of DOX reaches 20 μg/mL, the cell survival rate under hypoxic conditions drops to 64.5 ± 0.6%. These results demonstrate that the DOX-loaded micelles have a strong killing capacity for breast cancer cells under hypoxic conditions.

### Cell Apoptosis

Finally, the cell apoptosis of micelles was determined in MCF-7 cells by TUNEL assay.

**Figure 5:**
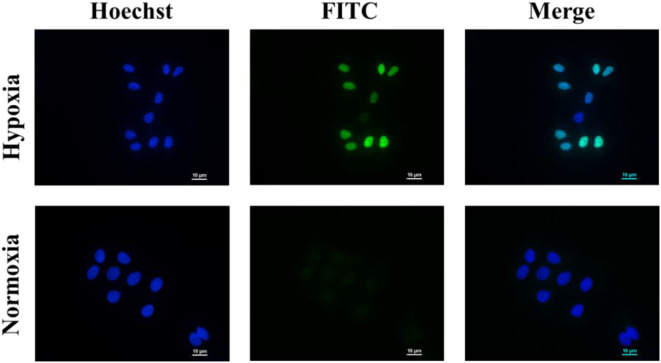
Cell apoptosis of PEGN/DOX under normoxic and hypoxic conditions. Scale bars are all 10 μm.

As shown in Figure 5, the nuclei of the apoptotic cells were stained green. After being incubated with PEGN/DOX for 24 h, the apoptosis rate of MCF-7 cells at the hypoxic condition was 74.57%, and no significant apoptosis of MCF-7 cells was detected in normoxic conditions, indicating that the DOX-loaded micelles could kill cancer cells more efficiently under hypoxic conditions, which is consistent with the cell uptake experiment.

## Conclusions

In conclusion, a hypoxia-responsive copolymer PEG-b-P(LGlu-NI) was successfully synthesized and self-assembled into micelles with DOX encapsulated in the hydrophobic core. The DLS results confirmed that the particle size of micelles was around 150 nm. Cell experiments show that DOX-loaded micelles have a stronger killing effect on tumor cells under hypoxic conditions, while the carriers have good biocompatibility. These results show that polymeric micelles have good potential of application in hypoxia-responsive drug release for enhancing cancer treatment.

## Data Availability Statement

The datasets presented in this study can be found in online repositories. The names of the repository/repositories and accession number(s) can be found in the article/supplementary material.

## Author Contributions

ZL and JL conceived and supervised the project. HF, DC, and FY performed all the experiments. BF and LJ wrote the manuscript. All authors read and approved the manuscript.

## Conflict of Interest

The authors declare that the research was conducted in the absence of any commercial or financial relationships that could be construed as a potential conflict of interest.
